# A Current View of Functional Biomaterials for Wound Care, Molecular and Cellular Therapies

**DOI:** 10.1155/2015/403801

**Published:** 2015-10-05

**Authors:** Francesco Piraino, Šeila Selimović

**Affiliations:** ^1^Institute of Bioengineering, School of Engineering, École Polytechnique Fédérale de Lausanne, 1015 Lausanne, Switzerland; ^2^American Association for the Advancement of Science, Washington, DC 20520, USA

## Abstract

The intricate process of wound healing involves activation of biological pathways that work in concert to regenerate a tissue microenvironment consisting of cells and external cellular matrix (ECM) with enzymes, cytokines, and growth factors. Distinct stages characterize the mammalian response to tissue injury: hemostasis, inflammation, new tissue formation, and tissue remodeling. Hemostasis and inflammation start right after the injury, while the formation of new tissue, along with migration and proliferation of cells within the wound site, occurs during the first week to ten days after the injury. In this review paper, we discuss approaches in tissue engineering and regenerative medicine to address each of these processes through the application of biomaterials, either as support to the native microenvironment or as delivery vehicles for functional hemostatic, antibacterial, or anti-inflammatory agents. Molecular therapies are also discussed with particular attention to drug delivery methods and gene therapies. Finally, cellular treatments are reviewed, and an outlook on the future of drug delivery and wound care biomaterials is provided.

## 1. Introduction

**Figure 1:**
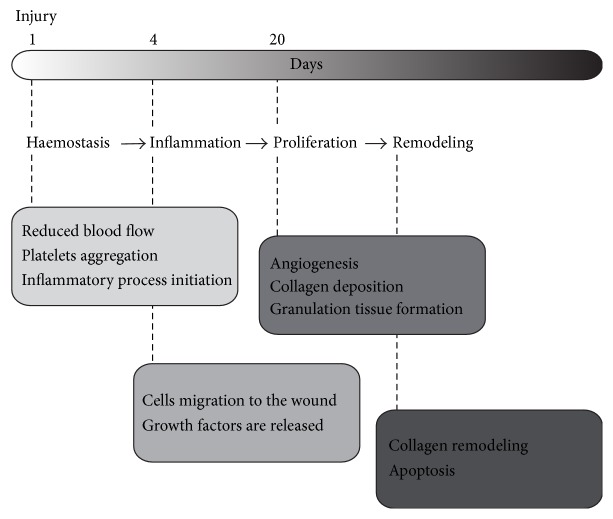
Overview of the wound healing process. The injury is immediately followed by hemostasis, which is characterized by reduced blood flow, platelet aggregation, and initiation of the inflammatory process. During the inflammation response, cells migrate to the wound and release growth factors. In a later stage of the wound healing process, the proliferation stage, angiogenesis is followed by deposition of collagen and the formation of granulation tissue. Finally, collagen fibers align in the remodeling stage and cells that have fulfilled their wound healing function enter apoptosis.

Tissue repair and wound healing are complex physiological processes in which the damaged tissue repairs itself after an injury, such as a superficial cut, internal bleeding, or excision of a tumor. The wound healing process is generally divided into the sequential, but partially overlapping phases (Figure 1) of hemostasis (clotting to stop bleeding), inflammatory response (removal of bacteria and tissue debris from the site of damage), proliferation (cell division to regenerate the tissue, angiogenesis, and matrix deposition), and remodeling (cellular apoptosis and matrix realignment in the newly generated tissue) [1]. The wound healing response involves direct cell-to-cell and cell-matrix communication, in addition to the indirect communication between different cell types* via* soluble molecules. These interactions can be enhanced or accelerated by the strategic delivery of hormones, hemostatic agents, anti-inflammatory drugs, angiogenesis-inducing compounds, and cell growth factors.

The specifics of a therapeutic approach depend on the type of wound and tissue properties. For example, wounds can be incisional (closed) or excisional (open), acute (result of a cut or a gunshot) or chronic (due to a long-term infection or underlying disease). In the latter case, the wound healing process is disrupted. Both superficial and internal wounds can be treated with a number of dressings, which range from topical pharmaceutical formulations as well as gauzes and synthetic dressings to modern materials like hydrocolloids, hydrogels and foams, and biomaterials. The type of wound dictates the choice of drug and drug delivery vehicle to aid the tissue repair process: a dressing might be chosen for its ability to absorb exudates from the wound or to degrade and release a biopolymer used in the proliferation stage. Another material might prove a better drug delivery vehicle, while yet another one would be better suited to encapsulating cell growth factors. Last, but not least, factors affecting the choice of treatment also include cost, ease of handling, and the ability to accelerate the healing process.

In this review, we focus on wound healing processes in adult tissue (as opposed to fetal tissue) and introduce a number of molecules that play key roles in these processes. We then discuss interactions between tissues and biomaterials, such as requirements for biomaterial-based dressings. Finally, we address molecular and cellular therapies for treatment of external and internal wounds.

## 2. The Natural Wound Healing Process

### 2.1. Hemostasis

Hemostasis involves a series of processes that work together to stop the bleeding from a wound. In intact blood vessels, endothelial cells secrete the coagulation inhibitor thrombomodulin and produce prostacyclin and nitric oxide to prevent aggregation of platelets [2]. In case of an injury to a blood vessel, endothelial cells switch to producing von Willebrand factor (vWF) in order to jump start hemostasis. Concurrent with this process is vasoconstriction in order to limit the amount of blood leaving the damaged vessel and is followed by the formation of a platelet plug that blocks the break in the vessel. The last step in hemostasis is the formation of a fibrin clot through production of plasma factor VII (FVII) [3, 4] and prothrombin. Fibrin is a type of collagen fiber and is produced around the platelet plug, anchoring it in place [5]. White and red blood cells become entrapped in the fibrin structure, a process called blood coagulation. Both the platelet plug and fibrin clot serve to seal the physical hole in the blood vessel until the tissue is healed.

### 2.2. Inflammatory Response

In the inflammation phase, interleukins, a type of cytokine, are activated. Interleukin 6 (IL-6) [6] stimulates macrophage activation and chemotaxis of monocytes, and interleukin 8 (IL-8) [7] encourages neovascularization and proliferation of neutrophils. The different types of leukocytes are responsible for countering pathogens and for degrading the damaged tissue and creating new, healthy tissue. The remaining stages of the wound healing response are particularly sensitive to an abnormal increase or reduction in leukocyte activity.

### 2.3. Proliferation

In the proliferation stage, macrophages and neutrophils release chemoattractants to draw fibroblasts to the wound site and enable synthesis and remodeling of the extracellular matrix (ECM) [8]. Cellular migration is aided by the production of hyaluronic acid (HA), which absorbs water and lends the tissue the ability to resist deformation [9].

### 2.4. Remodeling

Collagen is the most abundant structural protein in the human ECM. Collagen Type I predominates and is upregulated by decorin in wound healing [10]. The production of disorganized and strongly cross-linked Type I collagen structures leads to fibrosis or scars, new tissue that is visually distinct from the surrounding, undamaged tissue.

Remodeling of the ECM is also facilitated by proteases such as tissue-derived inhibitors (TIMPs) and matrix metalloproteinases (MMPs), for example, collagenase [8]. The formation of scars is marked by increased TIMP-1 [11] and TIMP-3 [12] activity. In general, scarring is accompanied by a lower MMP to TIMP expression ratio, as a slow collagen turnover leads to increased protein accumulation. Plasminogen is another protease central to wound repair [13]. When activated to plasmin, it promotes fibrinolysis, preventing the fibrin clot from growing and ultimately degrading it.

Transforming growth factor-betas are a family of cytokines involved in many parts of the cell cycle: cell growth, proliferation, differentiation, and apoptosis [14]. They serve as chemotactic for fibroblasts, stimulating production of collagen Type I. Concurrently, TGF-*β* also decreases MMP expression, leading to the accumulation of collagen. In case of an injury to the tissue, expression of TGF-*β* is upregulated by signaling factors like decorin, fibromodulin, and hypoxia-inducible factor-1-alpha (HIF-1-alpha). With respect to cutaneous wounds, TGF-*β* signaling in keratinocytes is reduced, leading to accelerated reepithelization of the wound [15].

Two additional growth factors that enhance fibrosis are the platelet-derived growth factor (PDGF) [16, 17] and fibroblast growth factor (FGF) [18]. PDGF serves as a fibroblast chemoattractant and its expression increases during the formation of fibrotic tissue. FGF is actually a collection of multiple cytokines such as keratinocyte growth factors, which are expressed more strongly in wound repair than in healthy tissue.

Lastly, a cytokine that signals endothelial cells to enter the mitotic stage is the vascular endothelial growth factor (VEGF) [19]. Noteworthy, the expression of VEGF is increased in nonfibrotic wounds compared to scar wounds.

## 3. Interactions between Tissues and Biomaterials

### 3.1. Functional Requirements of Wound Repair Biomaterials

Hydrogel-based dressings for skin wounds provide a barrier between the wound and the external environment, thus preventing infection and absorbing exudates (such as water, plasma, and red blood cells). Similarly, biomaterials used for internal wounds should repel or damage microbes and other infectious agents, be hydrophilic and sufficiently porous to absorb exuded liquids, and/or have a large enough swelling factor to fill any voids within the damaged tissue. In order to prevent an inflammatory response to the hydrogel itself, the material should be biologic and degradable on a time scale comparable to the wound healing process (on the order of days) [20, 21]. In addition, hydrogels can serve as delivery vehicles for drugs and other wound healing compounds and can be manipulated to allow for controlled release of these components in both space and time [22, 23]. Hence, the cellular response at a wound site can be controlled and significantly accelerated by providing hemostatic, immunomodulatory, antibiotic, angiogenesis, and cell growth agents as regulated by hydrogel carriers.

Medical dressings are engineered to fulfill one or several functions: stem bleeding by helping to seal the wound, absorb plasma, blood, and other exuded fluids, debride the wound by removing foreign objects from the site, protect the affected area from pathogens, and aid the granulation or improve epithelization. Depending on the goal of the treatment, the dressing can be designed to control the moisture content of the wound, prevent an infection, or maintain the optimal microenvironment (such as pH and temperature). The treatment goal then dictates the design parameters of the biological dressing, such as hydrophilicity (dressing can be either hydrophilic or hydrophobic to control the rate of fluid passage from the wound), porosity and swelling ratio (to allow an encapsulated drug to diffuse into the wound), and degradation (to release the biomaterial into the wound and aid the tissue regeneration).

More specifically, advanced wound therapies focus, among others, on either preventing ECM damage by delivering specific proteins to the wound site or enabling ECM synthesis through various growth factors or autologous proteins. For example, ECMs contained in hydrogel-based dressings can allow cellular adhesion and thus aid tissue regeneration.

The goals listed above can be achieved with a number of bioactive hydrogels, such as those based on collagen, HA, chitosan, alginate, or elastin, or also multiarm (poly)ethylene glycol (PEG) precursors. For example, certain molecules from the ECM can be tethered to hydrogels such as PEG to render them bioactive [24]. Such bioactive hydrogels have been shown to be cytocompatible and do not provoke significant inflammatory responses, but they nonetheless provide useful physical and chemical characteristics to support the tissue regeneration process [20, 25]. For example, alginate-based dressings usually have large swelling ratios and are capable of absorbing large exudate volumes in wounds [26]. In addition, they can also be applied to dry wounds after a treatment with saline. Collagen, being the main structural protein in various connective tissues, is a prime candidate for dressings providing ECM structures [27]. Next, chitin and chitosan are known for their adhesive, but also antibacterial and fungicidal properties. This makes both polymers useful for wound dressings in any form ranging from fibers and membranes to larger scaffolds and hydrogels [28, 29]. Hyaluronan, another chief component of the ECM, is also associated with ECM remodeling and contributes to cell proliferation [9, 30]. For example, some dressings used for chronic wound treatment utilize hyaluronan-based scaffolds, with fibronectin connected to the protein to aid the migration of cells into the wound. Finally, elastin is a load-bearing and a greatly stretchable protein in connective tissue that helps, for example, skin reestablish its barrier function after an injury [31]. Elastin also helps induce ECM synthesis, cell migration, and production of proteases.

Aside from their native properties, various biomaterials can be used to deliver functional molecules to the wound site, including therapeutics. For example, alginate and PEG are easily functionalized using chemical conjugation methods [32], while poly(lactic-*co*-glycolic acid) (PLGA) based materials [33, 34] can be used for controlled release of molecules embedded into the biomaterial scaffold. Stromal cell-derived factor (SDF, a set of cytokines that activate leukocytes), VEGF, and PDGF can be encapsulated into PLGA capsules and released over time to induce endothelial cell migration and vessel formation or to stabilize blood vessels and ultimately induce angiogenesis. As a result, PLGA finds applications in sutures and implants due to its degradation properties.

By functionalizing biomaterials or tuning their physical properties, it is possible to design novel wound healing materials with optimal antibacterial, anti-inflammatory, and adhesive properties. To induce desired hemostatic processes, glycoproteins such as the vWF can be encapsulated into the biomaterials scaffolds [35]. In addition, anti-inflammatory molecules can be delivered to the wound site encapsulated in calcium alginate gels [26]. Finally, the antibacterial properties of some biomaterials dressings can be boosted by encapsulating antibacterial agents such as vancomycin [36] and amoxicillin [37] into the scaffolds and hydrogels.

### 3.2. Biomaterials Interactions at the Surface

The surface interaction between biomaterials and the damaged tissue occurs at various length scales, from the organ (millimeters to centimeters) and tissue scale (millimeters) to the scale of individual cells (micrometers) and proteins (nanometers) [38, 39]. A tissue or part of an organ can be in contact with a biomaterial-based ECM or dressing for weeks, and in the case of organs even up to months or years, and the interactions are based on physical contact, tissue ingrowth, and chemical bonding. The smaller the tissue component, the shorter the interaction time: individual cells interact with a biomaterial for days or weeks* via* integrin, while individual proteins (such as glycosaminoglycans (GAGs)) interact through secondary bonding and hydrophobic interactions on time scales as short as seconds and minutes. Among the physical and mechanical interaction mechanisms are the entanglement of macromolecules and interdigitation of the ECM with the physical biomaterial structure, for example, pores. The main chemical type of interaction is ionic, covalent, or metallic, and it can be accompanied by hydrogen bonding and van der Waals and hydrophobic interactions.

Biomaterial surfaces can induce changes in cell phenotype, including the cell morphology, development, or biochemical properties. Knowing this, various properties of biomaterial-based dressings can be engineered to facilitate an optimal tissue regeneration rate. For example, the pore size of a scaffold can serve to regulate the migration speed of cells: the smaller the average pore size, the lower the fibroblast migration speed in collagen-GAG scaffolds [40]. Nonetheless, it has been shown that other cells, such as prostate cancer cells, migrate faster than fibroblasts through the same scaffold. In addition, a decrease in pore diameter is linked to an increase in the specific biomaterial surface, offering a greater density of binding sites for cell attachment.

Using collagen as an example, one can delay the biomaterial degradation by tuning the degree of crosslinking, grafting GAG molecules onto collagen fibers, and preserving the native polymer structure (or avoiding the premature degradation of collagen into gelatin) [41]. Noteworthy, it has been showed that the melting of the quaternary collagen structure can reduce thrombosis by inhibiting platelet clotting and decrease the inflammatory response [42]. In general, biomaterial scaffolds (regeneration templates) can lose their activity if their chemical composition, quaternary protein structure, pore size, and rate of degradation are outside of the optimal range.

## 4. Molecular Therapies

### 4.1. Drug Delivery Methods

Most therapeutics are administered orally, in the form of aerosols, liquids, capsules, or tablets, such that they enter the circulatory system through the gut lining (or, in the case of aerosols, through the lung). Intravascular and intramuscular injections are another often used transportation method that allows the circulatory system to transport the drug throughout the body, including to the wound site [43–46]. In the case of external or open wounds, dressings can also contain bioactive agents that are embedded into the biomaterial scaffold. In general, drug delivery devices rely on their specifically designed physical and chemical properties (described earlier) to transport the drug and ultimately deliver the appropriate bioactive agent at the appropriate time to the site of interest, the wound site. The therapeutics or bioactive agents range from small-molecule drugs to antibodies, proteins, plasmid DNA, and oligonucleotides. Noteworthily, drug delivery devices do not only deliver molecules to the wound site, but can also control the presentation of the encapsulated agents. One example is the use of genetically modified cells that are engineered to express various growth factors or cytokines, a common approach in gene therapy.

### 4.2. Gene Therapy

Gene therapy is a research field focusing on genetic modification of cells for therapeutic purposes. This discipline has developed approaches for permanent or transient cellular transformation. The concept of gene therapy arose during the 1970s when Friedmann and Roblin proposed guidelines for the gene therapy in humans [47]. Since then, gene therapy for wound treatments has mainly been represented in experiments with animal wound models [48]. Nevertheless, the development of new gene therapy protocols for treatment of a wide array of monogenetic as well as multifactorial diseases has continued and lately Margolis and coworkers reported the results of the first clinical trial in humans for gene therapy in wound healing [49].

The cellular events driving regenerative processes are strongly regulated by complex molecular mechanisms. Manipulation of these mechanisms at the genetic level offers several advantages over exogenous application of substances.

**Figure 2:**
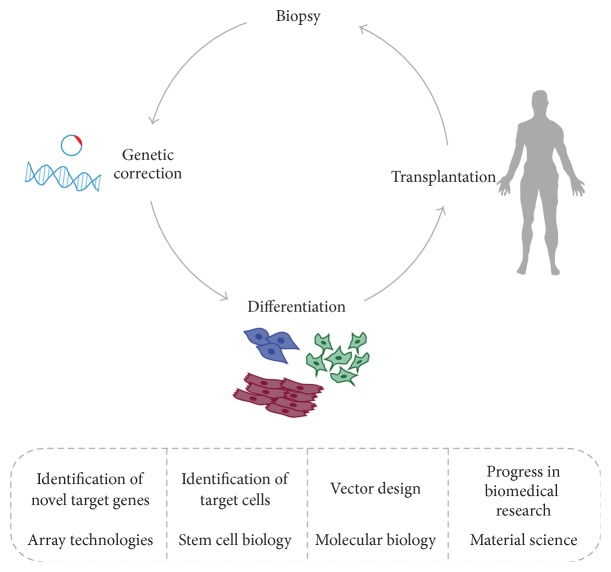
Genetic modifications for patient-specific therapy. This technique can be divided into three main phases. First, a biopsy is performed and cells are expanded* in vitro*. Second, a gene is introduced into these cells. Finally, the genetically engineered cells are transplanted to the patient. The dashed box reports the different research fields involved in this type of approach.

*In vivo* or* in vitro* methodologies can be employed to convey genes to the tissue of interest [50]. In the first approach, the cells are isolated, cultured, and genetically modified* in vitro* and subsequently implanted into the tissue as illustrated in Figure 2. Although laborious, the method enables selective gene transfer to the specific targeted cell type. The efficiency of the transfection can be quantified and the risk of systemic contamination can be reduced by automation. Furthermore, the risks of detrimental effects related to systemic vector administration are avoided. The second approach includes direct delivery of the gene to the targeted cell. Vectors may be directed to the site of tissue repair by topical application, injection or carried by biomaterial scaffolds. The limitations of this method are the low transfection efficiency and the lack of complete specificity. The selection of an appropriate delivery system is vital for effective gene therapy. The cells must be able to take up the transgene and express the product within a specific time period in a required amount. Moreover, any gene delivery technology should be nontoxic, atraumatic and should not evoke immune responses [51].

The various delivery systems can be categorized into biological, physical, and chemical techniques. Biological methods are additionally classified into viral and nonviral methods. Viral methods use viruses as vectors for delivery of the genetic material to the recipient cell. Nonviral biological vectors may be bacteria, bacteriophages, virus-like particles, or biological liposomes. The nonbiological methods for gene delivery may be physical and chemical. The transgenes to be delivered are incorporated into closed circular DNA molecules called plasmids. Electroporation, ultrasound, needle injection, or hydrodynamic delivery engages a physical force to deliver the transgene into the cell. Chemical approaches use natural or synthetic substances as transgene carriers.

The increased understanding of the complex molecular mechanisms that regulate cells participating in tissue repair has laid a foundation for therapeutic interventions with the purpose of enhancing wound healing. Such therapies could be applied to increase the rate of wound healing and enhance the quality of newly formed tissue in wounds complicated by delayed or insufficient healing. Furthermore, molecular mechanisms could be manipulated to prevent excessive scarring in fibrotic conditions such as hypertrophic scars and keloids. In this context, gene therapy offers several advantages over direct administration of peptide factors. Peptides delivered to the wound environment are highly susceptible to proteolytic degradation, lowering the effective dose. Furthermore, sequestration by the wound matrix may prevent binding to receptors at the surfaces of cells. Gene therapy allows sustained and regulated secretion of factors in their proper spatial and temporal context. This aspect is particularly important as growth factors may have different effects depending on cell type, concentration, and other simultaneous signals from soluble factors, adhesion molecules, and matrix components.

Growth factor genes have been widely used in experimental gene therapy to enhance wound healing. The most commonly used approach has been to overexpress genes that stimulate reepithelialization [52] or angiogenesis [53–56].

Gene therapy has shown great promise in the experimental setting as a way to enhance wound healing. Clinical trials are underway to assess the safety of various gene therapy protocols in human subjects. Further development will, however, be needed to bring gene therapy from the experimental setting to established clinical practice. To achieve this, several issues will have to be addressed. Technologies for gene delivery will have to be further improved to achieve cell-type specific and efficient transformation without eliciting an immune response or provoking toxic reactions. Gene therapy in wound healing should not be limited to the overexpression of single growth factors with the aim to accelerate tissue repair. The identification of new target genes will be an important step to increase the possibilities for quantitatively as well as qualitatively enhanced wound healing. In particular the targeting of genes coding for intracellular factors may become a way to increase the therapeutic specificity. Furthermore, simultaneous delivery of several transgenes, and at different time-points, will increase the possibilities to stimulate several events, acting in concert to enhance healing.

## 5. Cellular Therapies

Usually wound management involves examining the cause of injury and letting the body to recover. The emergence of regenerative medicine coupled with an increased understanding of the cellular and biochemical factors involved in wound repair has provided new therapeutic options which aim to alter the wound microenvironment facilitating regeneration.

### 5.1. Biomaterials for Cell Therapies

Polymers originating from biological sources are usually divided into nucleotide, protein and poly(amino acid), and polysaccharide and poly(hydroxyalkanoate). Biopolymers have been investigated for the preparation of biomaterials for a range of applications. However, biomaterial scaffolds from biopolymers often require improved mechanical properties, control of porosity, and optimized processing for practical use, regenerative medicine. Many studies concerning the preparation and application of biopolymer-based scaffolds have been conducted.  Table 1 reports references for some biopolymers.

The development of cellular therapies for wound repair dates back more than 30 years with the publication of the first successful protocols for the culture of keratinocytes [57]. Eventually, small sheets of cells were developed [58]. Since these early efforts, a better understanding of the wound healing process, coupled with advances in cell culture techniques and the development of* in vitro *bioreactor systems, has led to the creation of more complex tissue engineered constructs.

Current constructs lack the functional sensory nerves. Melanocytes have been used to repopulate burn scars and for the treatment of vitiligo [59], but they have yet to be included in a commercial skin substitute. A significant limitation has been the limited viability of allogeneic cells used in the majority of tissue engineered skin equivalents and the high cost and limited shelf life associated with using autologous cells. Skin needs to be capable of regeneration, growth, and adaptation to the wound site. Cell persistence is therefore an important consideration in developing new skin substitutes. While the objective of cellular therapies is to create a substitute for skin* in vitro* that can integrate into the engraftment site* in vivo*, an alternative approach is to engineer a biocompatible, resorbable matrix that can recruit the native tissue cells to the injured site and facilitate wound healing. Control of the wound microenvironment is a critical aspect of this wound healing approach. As has been demonstrated with the currently available living skin equivalents, the delivery of ECM components and growth factors, and not necessarily the delivery of cells, to the site of injury appears to have the most beneficial effect [60]. Recent discoveries in stem cell biology and regenerative medicine have emphasized a strategy that may be more productive than the traditional cell-centric approach. By providing the correct microenvironmental niche, it may be possible to promote wound regeneration* in situ*.

### 5.2. Stem Cells for Wound Healing

Treatment of nonhealing wounds has remained difficult in spite of the better understanding of pathophysiologic principles. Early data suggest the use of multipotent stem cells in order to accelerate wound healing. Until recently, research has mostly been focused on bone marrow-derived mesenchymal stem cells; still adipose-derived stem cells (ADSCs) and those derived from hair follicles gain more and more interest for potential application for the restoration of various injured tissues.

Stem cells are considered able to differentiate and have a lengthy self-renewal capacity [61, 62]. These properties have raised the hope that human embryonic stem cells (hESCs) can be valuable for the treatment of various injuries [63]. Notwithstanding their exceptional potential, the use of embryonic stem cells remains debated in scientific and political circles. To circumvent ethical issues, Yamanaka and coworkers created pluripotent somatic cells by direct reprogramming and generated pluripotent stem cells from human somatic cells that were analogous to hESCs [64, 65]. Therefore, stem cells derived from adult sources could potentially be similar to embryonic stem cells. The opportunity to regenerate injured tissues is opening the way to new cures that require strict assessment in preliminary clinical trials.

Encouraging findings from stem cell-based treatments in postinfarction myocardial repair [66] have led to the application of similar strategies in order to treat skin wounds [67–70]. Nonetheless, stem cells use seems beneficial over diffusible factors because stem cells can interact with their wound microenvironment [71]. Conversely, despite the above-mentioned improvement in wound healing using various stem cell lines, several issues need to be pondered before administering stem cells to patients. For example, stem cells functionality decreases with age; thus, older patients may not present the perfect population as donors [72]. Also, the risk of immunological rejection upon transplant or transfusion must be considered in case of using stem cells from allogenic sources. The mode of stem cells delivery is another frequently discussed issue. Ideally, stem cells should keep their multipotency until administered in order to boost engraftment [70].

In conclusion, wound healing necessitates a sound combination of cell migration and proliferation, in addition to ECM deposition, angiogenesis, and remodeling. A variety of sources have been exploited to isolate stem cells for wound healing. Still, additional efforts are necessary in order to solve the many questions on stem cells' clinical application.

## 6. Conclusion and Outlook

Over the past decades, extraordinary advances and improved understanding in medicine, materials science, and engineering have led to great achievements in drug delivery and wound healing. In addition, microfluidic technologies have shown unparalleled advantages for biomaterials synthesis and for design of drug delivery systems based on cells. Furthermore, generation of concentration gradients of biochemical molecules [73] and within hydrogels [74, 75] plays a key role in tissue morphogenesis as well as in wound healing, bacterial invasion, and immune response. Microfluidics can additionally offer integrated structures to resemble the* in vivo* cellular environment. The transition from 2D to 3D cell culture has developed as a growing number of studies have established meaningful changes in the morphology, migration, differentiation, and viability of cells between 3D and 2D. Hence, efforts have been made to produce 3D platforms to mimic the* in vivo* microenvironment [76–78]. Based on these events, we expect that in the future integrated microfluidic devices for wound care will be able to monitor all the vital signs of the healing process, such as oxygen levels and temperature, and make adjustments when needed and communicate the information to health professionals on- or off-site [79].

Challenges, however, still exist. For example, gene delivery technologies will require further improvement to enable cell-type specific and efficient transformation without provoking an immune response. Ideally, both cellular and acellular treatments should enable delivery and spatiotemporal control of multiple molecules at the wound site without requiring intervention from a medical professional. Finally, the identification of new target genes as well as new biomaterial compounds with multiple tunable characteristics will be vital to a major step forward in wound healing treatments. Thus, future investigation in this path will be of tremendous relevance for translational medicine and therapeutics applications.

## Figures and Tables

**Table 1:** A selection of biopolymers used in wound healing.

Biologically derived polymers	References
Poly(hydroxyalkanoate)s	[80]
Poly[(R)-3-hydroxybutyrate]	[81–83]
Worm silk	[84]
Spider silk	[85]
Collagen	[27, 41, 42, 86]
Elastin	[31, 87–90]
Resilin	[91–93]
Keratin/chitosan	[94]
Cellulose	[95]
